# Enhanced diabetes prediction using skip-gated recurrent unit with gradient clipping approach

**DOI:** 10.3389/fendo.2025.1601883

**Published:** 2025-08-26

**Authors:** Suhas Kamshetty Chinnababu, Ananda Babu Jayachandra, Swathi Holalu Yogesh, Mohamed Abouhawwash, Doaa Sami Khafaga, Eman Abdullah Aldakheel, Vinaykumar Vajjanakurike Nagaraju

**Affiliations:** ^1^ Department of Information Science and Engineering, Malnad College of Engineering, Hassan, India; ^2^ Visvesvaraya Technological University, Belagavi, India; ^3^ Channabasaveshwara Institute of Technology, Tumkur, Karnataka, India; ^4^ Department of Computer Science and Engineering (Artificial Intelligence and Machine Learning), Malnad College of Engineering, Hassan, India; ^5^ Department of Industrial and Systems Engineering, King Fahd University of Petroleum and Minerals, Dhahran, Saudi Arabia; ^6^ Interdisciplinary Research Center of Smart Mobility and Logistics (IRC-SML), King Fahd University of Petroleum Minerals, Dhahran, Saudi Arabia; ^7^ Department of Computer Sciences, College of Computer and Information Sciences, Princess Nourah bint Abdulrahman University, Riyadh, Saudi Arabia; ^8^ Department of Artificial Intelligence and Machine Learning, Navkis College of Engineering, Hassan, India

**Keywords:** deep learning, diabetes mellitus, gradient clipping, machine learning, long-term dependencies, skip-gated recurrent unit

## Abstract

Diabetes mellitus is a metabolic disorder categorized using hyperglycemia that results from the body’s inability to adequately secrete and respond to insulin. Disease prediction using various machine learning (ML) approaches has gained attention because of its potential for early detection. However, it is a challenging task for ML-based algorithms to capture the long-term dependencies like glucose levels in the diabetes data. Hence, this research developed the skip-gated recurrent unit (Skip-GRU) with gradient clipping (GC) approach which is a deep learning (DL)-based approach to predict diabetes effectively. The Skip-GRU network effectively captures the long-term dependencies, and it ignores the unnecessary features and provides only the relevant features for diabetes prediction. The GC technique is used during the training process of the Skip-GRU network that mitigates the exploding gradients issue and helps to predict diabetes effectively. The proposed Skip-GRU with GC approach achieved 98.23% accuracy on a PIMA dataset and 97.65% accuracy on a LMCH dataset. The proposed approach effectively predicts diabetes compared with the existing conventional ML-based approaches.

## 1 Introduction

Diabetes mellitus (DM) is a chronic metabolic disease which affects the human body by impairing its ability to convert blood sugar to energy (1). People diagnosed with diabetes are unable to regulate their blood sugar level in the body, which resulted in high levels of blood pressure and sugar (2). If diabetes is not detected, diagnosed, and treated in the early phases, it causes life-threatening diseases like kidney failure, diabetic retinopathy, and various cardiovascular diseases (3). Despite the advances in the medical field in recent times, diabetes remains challenging in many societies (4). Therefore, it is crucial to design intelligent systems which provide support to medical personnel in the diagnosis of diabetes and decision-making (5). The classical lab tests that depend on diabetic diagnosis approaches are expensive and time-consuming (6). Generally, clinicians consider an approximate diagnosis and prediction of diabetes mellitus by considering fasting blood sugar or random blood sugar tests (7). The glycated hemoglobin (HbA1c) test was implemented to the public in 1980 for diagnosing diabetes in patients. This test analyzes a percentage of blood sugar attached to hemoglobin for over 3 months (8). This test process is complex, is time-consuming, and requires medical professionals and particular equipment for such to be performed (9).

Type 2 DM (T2DM) is a general diabetes category, and it is categorized as hyperglycemia because of insufficient insulin generation in the human body (10). Recently, numerous diabetes detection approaches have been developed by researchers (11). Many researchers have used datasets that contain lab-test-based medicine indicators for method validation and training process (12), but these prediction approaches considered extensive lab-test-based measurements to diagnose diabetes. However, there is an increasing demand for primary diagnostic solutions that do not depend on measurements of lab test (13). Hence, the research on anthropometric features and its impacts on diabetes prediction approaches has been performed (14). In data-driven diabetes detection solutions, machine learning (ML) has become a popular choice because of its classification capability, which processes statistical techniques without requiring much computation power (15).

The recent developments in diabetes prediction have majorly used ML, an ensemble of learning strategies to enhance the diagnostic accuracy and generalization ability. Simaiya (16) presented the multistage ensemble model integrating classifiers across layers to improve prediction performance on PIMA dataset. Thakur et al. (17) suggested for the model to focus on COVID-19 context, combining advanced feature engineering and ensemble algorithms to enhance prediction accuracy, highlighting the significance of early diagnosis in vulnerable populations. Kaliappan et al. (18) analyzed different datasets by ML algorithms with explainable AI tools such as SHAP and LIME, highlighting the part of model interpretability and data diversity in reliable diabetes prediction. Abousaber et al. (19) addressed the data imbalance issues through integrating ensemble models with oversampling algorithms, obtaining robustness across PIMA and real-world clinical datasets. However, the ML-based algorithms were unable to capture the long-term dependencies in diabetes, and certain research skipped the feature selection process. These drawbacks reduce the performance of diabetes predictions, and to overcome these drawbacks, in this article, the deep learning (DL)-based algorithms were developed, which captures the long-term dependencies effectively. In clinical settings, understanding the features that contribute to the model’s prediction is essential to enable effective decision-making. Hence, this research developed the model for accuracy and also for interpretability in feature level. The significant contributions of the article are described below:

- The skip-gated recurrent unit (Skip-GRU) with gradient clipping (GC) approach is developed, which captures the long-term dependencies in data and mitigates the exploding gradients issue during the training process. This allows the model to effectively predict diabetes with high classification performance.
- The random spiral flight strategy (RSFS)–marine predator algorithm (MPA) is developed for the feature selection process, which selects only the appropriate features from the entire feature subset and helps to enhance the classification performance.
- The integration of feature-skipping mechanism in the Skip-GRU architecture allows the jump probabilities to dynamically control the incorporation of input features. Features with high predictive contribution (e.g., glucose, BMI, HBA1C) are consistently retained across predictions and less significant features are skipped, which minimizes noise. By aligning this learned feature importance with LIME-based visual explanations, the model provides transparency of important features.

This research paper is organized as follows: Section 2 analyzes the different ML and DL approaches. Section 3 provides the process of diabetes prediction with detailed explanation. Section 4 analyzes the performance of the proposed method and presents results with comparison, and Section 5 concludes the article.

### 1.1 Research question and hypothesis

Question—Can the integration of Skip-GRU with GC and probabilistic feature selection improve the accuracy, interpretability, and generalization of diabetes prediction models across different clinical datasets?

Hypothesis—Integrating Skip-GRU with GC and feature selection will effectively improve the prediction performance and interpretability in clinical diabetes datasets compared to traditional models.

## 2 Literature review

This section analyzed the different ML- and DL-based approaches for diabetes prediction on PIMA, LMCH, and other standard and collected datasets. These approaches are described with their process, advantages, and drawbacks.

Saeed (20) suggested various ML classifiers like gradient boosting, AdaBoost, decision tree (DT), and Extra Tree classifiers for detecting chronic diabetes disease. These methods analyzed PIMA Indian Diabetes dataset (PIMA) and Behavioural Risk Factor Surveillance System (BRFSS) datasets for classifying the patients with positive or negative diagnosis. The suggested ML algorithms effectively predict diabetes, but the ML algorithms cannot capture the long-term dependencies in datasets like glucose levels.

Olisah et al. (21) presented the framework including Spearman correlation and polynomial regression to select features and impute missing values. Various supervised ML algorithms like random forest (RF), support vector machine (SVM), and twice growth deep neural network (2GDNN) were used for classification. The methods were optimized using hyperparameter tuning through grid search and k-fold validation, which were analyzed for its effectiveness to address the prediction issue, but the method does not scale the value in uniform range that reduces the classification performance.

Reza et al. (22) introduced the improved non-linear kernel for the SVM method for enhancing the type 2 diabetes classification. The new kernel utilized the radial basis function (RBF) and RBF city block kernels which enabled the SVM to learn difficult decision boundaries and adapted intricacies. For addressing missing values and outliers, imputation was performed using the median, which ensures the integrity of the dataset. The class imbalance problem was mitigated by leveraging the robust synthetic-based over-sampling technique. However, the SVM approach was unable to capture the long-term dependencies in data, which was crucial for diabetes prediction.

Patro et al. (23) developed the data modeling which depended on correlation measures among features and was utilized for processing the data efficiently to predict diabetes. The standard available Pima Indians Medical Diabetes (PIMA) dataset was used to verify the effectiveness of the developed methods. The developed method predicted diabetes in the early phase and improved the accuracy, but the method does not address the issue of exploding gradients during the training phase, which affects the classification performance.

Dharmarathne et al. (24) implemented the ML interpretation technique called Shapley Additive Explanations (SHAP). Every method exhibited commendable accuracy in detecting patients with diabetes, with the XGB method showing a little edge. By using SHAP, research on the XGB method provided in-depth insights into the reason behind its prediction at a granular phase. The XGB method and local explanation of SHAP were combined to interface the predictions of diabetes in patients. The implemented method reduced the risks associated with diabetes by enhancing awareness, but the method did not consider the feature selection phase, so the whole feature subsets were fed to the classifier, which minimizes the classification performance.

Ejiyi et al. (25) suggested data augmentation and imputing the missing values as the preliminary phases. This method utilized the SHAP for extracting the feature significance and many significant features to fit the Extra Tree (ET), RF, Adaboost, and Xgboost techniques. The SHAP shows that glucose has a specific feature which contributes to many diabetes predictions when integrated with body mass index (BMI) and age. The suggested method effectively predicted diabetes with high performance, but it had less classification performance due to the values in the dataset that were not scaled uniformly.

Bhaskar et al. (26) developed the deep hybrid correlational neural network (CORNN) for detecting diabetes in patients. A few modifications were made to the network layout to enhance the classification accuracy of learning methods. The developed method has enhanced accuracy when compared with non-invasive methods. The CORNN method efficiently classified diabetes with high classification accuracy, but the developed method suffered from the issue of exploding gradients, which leads to less classification performance.

Ganie et al. (27) introduced the five boosting techniques for predicting diabetes on the PIMA diabetes dataset. The dataset was acquired from the University of California Irvine (UCI) ML repository and included numerous significant clinical features. Experimental analysis of data was utilized to identify the data characteristics. Additionally, upsampling, normalization, feature selection, and tuning of hyperparameters were assigned for predictive analysis. The introduced method effectively handled the features in the dataset and improved the performance, but the method does not impute the missing values in the dataset, which leads to less classification performance.

Alnowaiser (28) developed the automatic technique to predict diabetes with concentration on appropriately dealing with missing data and enhancing accuracy. The developed method utilized the K-nearest neighbor (KNN) imputed features with the Tri-ensemble voting classifier method. The developed ensemble method effectively handled the missing values and improved the accuracy but used ML algorithms that cannot capture the long-term dependencies for diabetes prediction.

Tasin et al. (29) implemented the semi-supervised method with XGB used for predicting the insulin features of the standard dataset. The ADASYN and SMOTE techniques are assigned for handling the issues of class imbalance. The ML classification techniques such as DT, SVM, RF, logistic regression (LR), KNN, and different ensemble methods for determining the technique provided good prediction outcomes. However, the method showed less classification performance due to classification errors in the performance.

The existing techniques have some drawbacks like the values in the dataset do not scale uniformly. Some methods do not impute the missing values and then skip the feature selection process. ML approaches were unable to capture the long-term dependencies and did not address the issue of exploding gradients. To overcome these drawbacks from the existing techniques, this article used the min–max normalization technique to scale the values uniformly, and the polynomial regression (PR) technique was used to impute the missing values in the dataset. Then, the RSFS–MPA-based feature selection algorithm is developed to select the relevant features, and then the classification is performed by using Skip-GRU with GC approach. This approach captures the long-term dependencies and mitigates the issue of exploding gradients with the help of the GC method during the training process. These processes help the model to predict diabetes effectively with high performance and accuracy.

## 3 Proposed method

**Figure 1:**
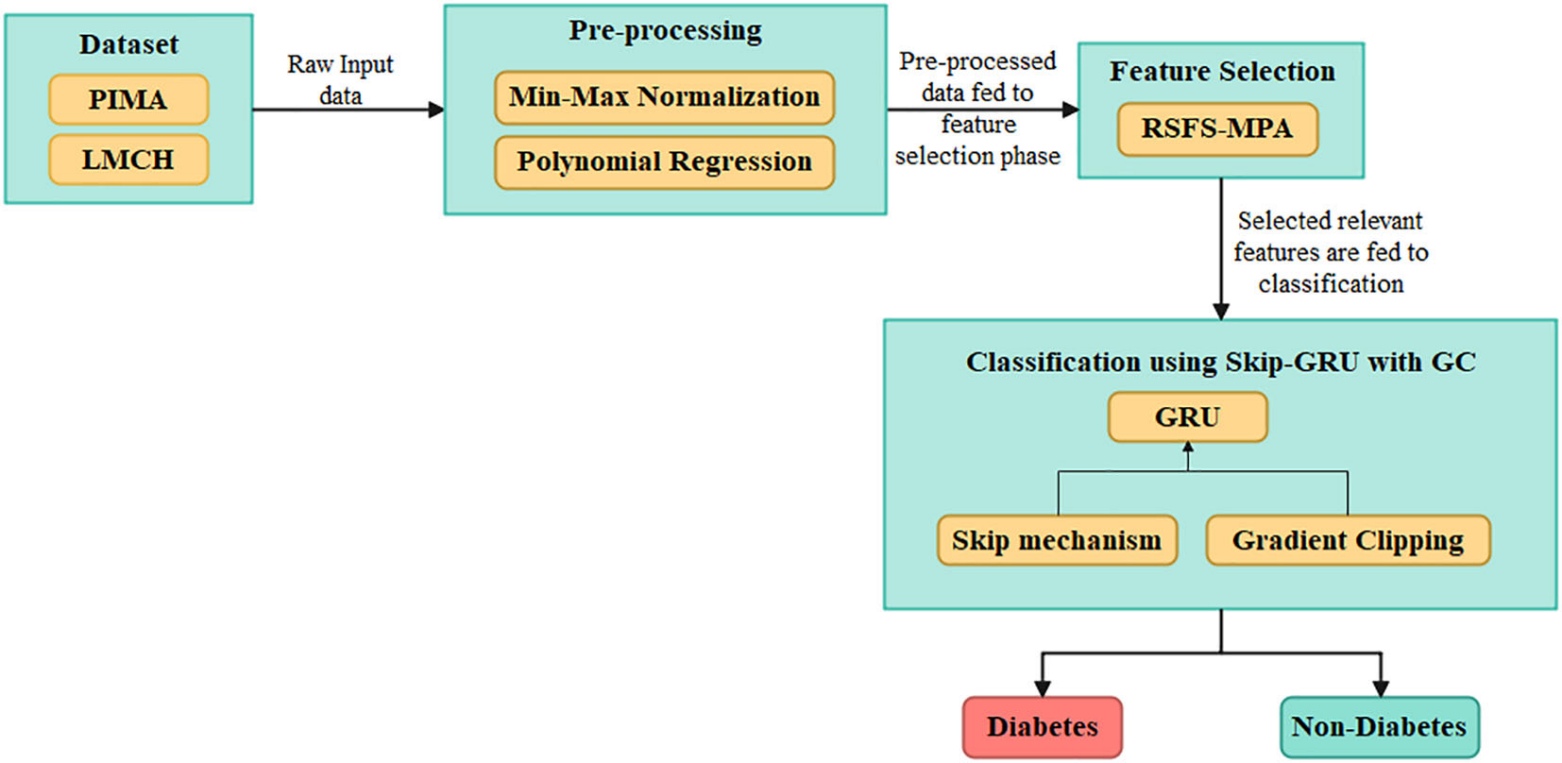
Overall process of the proposed Skip-GRU with GC model for diabetes classification using clinical datasets.

The efficient DL-based algorithm is developed in this article to predict diabetes effectively. The datasets used for the diabetes prediction are PIMA and LMCH. Then, the values in the dataset are pre-processed by using the min–max normalization that scales the data into (0,1) range, and the polynomial regression (PR) technique is used for imputing the missing values in data. Next, by developing the RSFS–MPA, the appropriate features are selected and then these are fed into the classification process. For classification, the Skip-GRU with GC approach is developed, which captures the long-term dependencies and effectively predicts diabetes. **Figure 1** describes the process of diabetes prediction.

### 3.1 Dataset

The dataset used in the article are PIMA Indian diabetes mellitus dataset and Laboratory of Medical City Hospital (LMCH) dataset. The detailed description of these datasets is explained in the following subsections.

#### 3.1.1 PIMA dataset

This dataset contains 768 instances, in which 268 patients are considered as a diabetic class and 500 patients are considered as a non-diabetic class (30). This dataset has eight attributes, and every patient is represented by these attributes. The eight attributes are glucose, blood pressure, pregnancies, skin thickness, body mass index (BMI), insulin, age, and diabetes pedigree function.

#### 3.1.2 LMCH dataset

**Figure 2:**
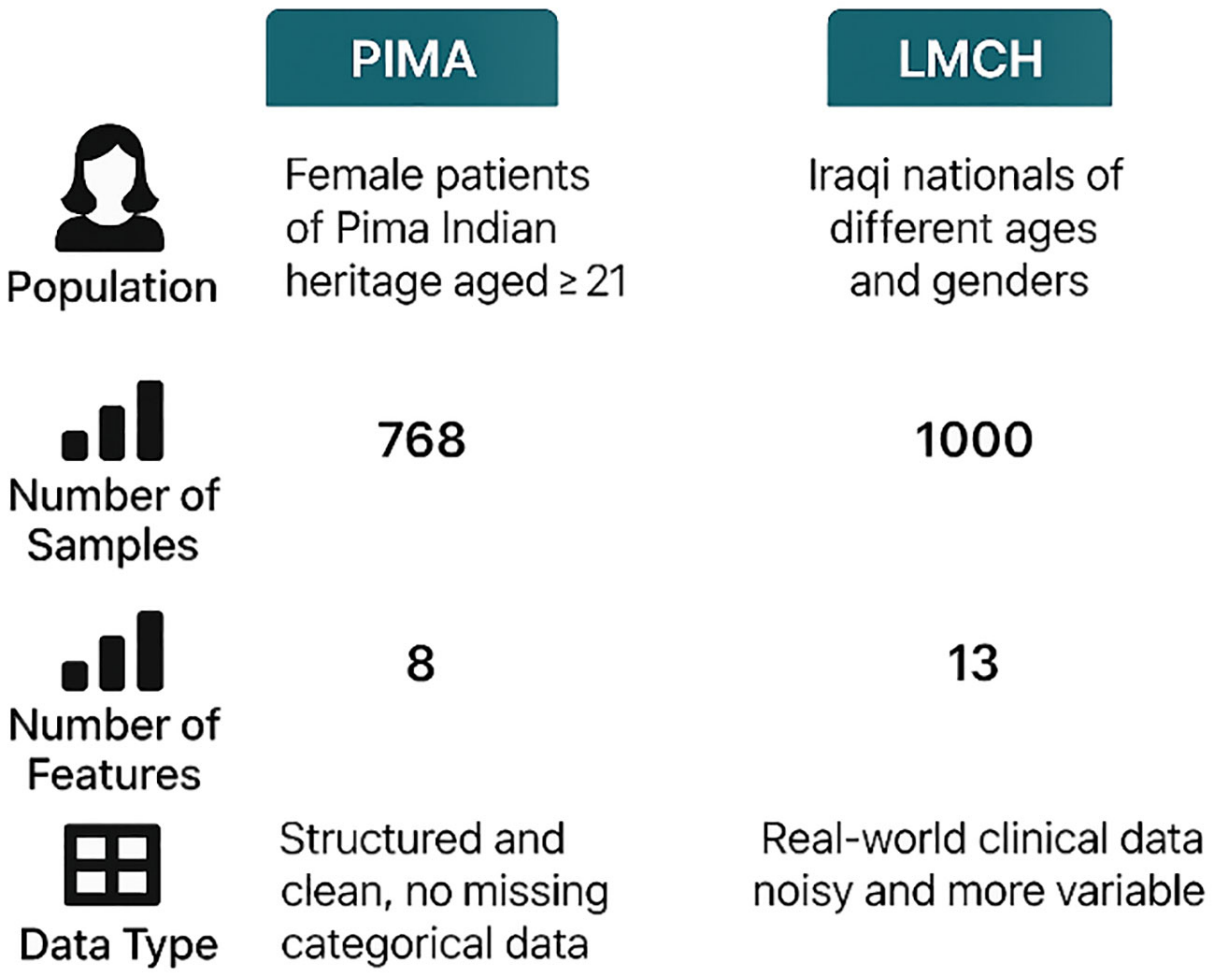
Characteristics of PIMA and LMCH datasets, including population demographics, sample size, number of features, and data types, highlighting differences on structure and complexity.

This dataset has 1,000 patients of Iraqi nationality gathered from LMCH, wherein 103 patients are considered as normal class, 53 patients are considered as prediabetes class, and 844 patients are considered as diabetes class (31). Every patient is represented by attributes like age, gender, blood sugar level, urea, BMI, creatinine ratio (Cr), and cholesterol (Chol) but also including total and fasting lipid profile, LDL, VLDL, HDL cholesterol, triglycerides, and HBA1C. The PIMA and LMCH datasets differ significantly in terms of population demographics, feature sets, and class distribution, which affect the model performance and generalization ability. **Figure 2** presents the characteristics of the PIMA and LMCH datasets.

### 3.2 Pre-processing

The values in the dataset are given as input for the pre-processing phase to normalize the data and to impute the missing values. The min–max normalization technique is used in this article to scale the data into 0 to 1, and then the polynomial regression (PR) technique is used to impute the missing values. A detailed explanation of the pre-processing techniques is described below:

Min–max normalization—It is the most common technique for scaling every class in the dataset, transforming every feature to have a minimum value of 0 and a maximum value of 1. The mathematical formula for min–max normalization is given as Equation 1.


(1) $${\text{x}}_{\text{scaled}}=\frac{\text{x}-{\text{x}}_{\text{max}}}{{\text{x}}_{\text{max}}-{\text{x}}_{\text{min}}}$$

In Equation 1 above, $x_{scaled}$ represents the normalized value, x represents the input value, and $x_{max}$ and $x_{min}$ represent the maximum and minimum value of attributes, respectively.

PR technique—Generally, in diabetes prediction, the mean and median are used to impute the missing values. Though this technique has increased the data bias and the multiple imputations of missing values (MICE) are used, it suffers from performance degradation due to the presence of non-linearities in predictor variables. This article used the predictive technique for imputing missing values by utilizing the PR with its non-linear regression. The input for missing value imputation is the values in a dataset. The process of missing value imputation is described as follows:

1. Initially, the percentage of missing values to every dataset is checked over the decision threshold of 5%. The decision is as follows: if a number of zero entries in a dataset is higher than 5%, the PR is used or else the entry is eliminated.
2. Then, data points are separated to non-zero and zero sets, where the non-zero set is utilized to testing and training when a zero set is predicted. The result is integrated with a non-zero set to develop a final dataset.

### 3.3 Feature selection

**Figure 3:**
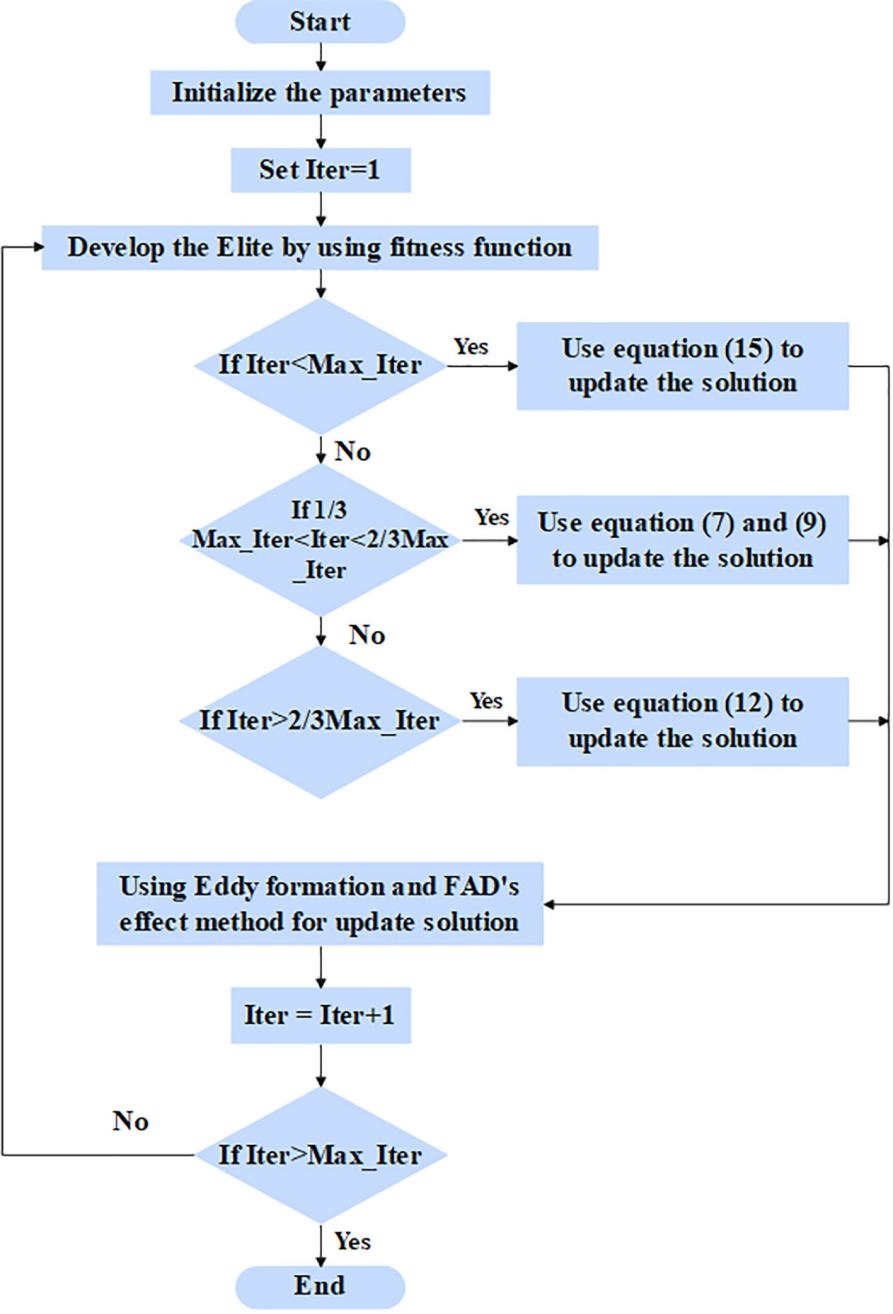
Flowchart for RSFS-MPA-based feature selection.

The pre-processed values are given as input to the feature selection phase to select the relevant features for classification. In this article, the RSFS–MPA is developed for the feature selection phase. The optimization-based algorithm is used, which searches the whole feature subset and chooses the best features out of them. The MPA is the nature-inspired swarm-based meta-heuristic optimization algorithm based on foraging behavior and meandering communications between predators and prey in oceanic ecological units (32). The RSFS is incorporated in the traditional MPA, which enhances the searchability of MPA for the feature selection process. The stopping criteria for the RSFS–MPA-based feature selection algorithm are set to 30 populations and a maximum of 100 iterations. The RSFS–MPA process includes initialization, exploration, exploitation vs. exploration, and exploitation, which are explained below. The flowchart for the RSFS–MPA is presented in **Figure 3**.

#### 3.3.1 Initialization phase

In the initialization phase, all of the populations are distributed uniformly in the search area, and its mathematical expression is given in Equation 2.


(2) $${\text{X}}_{0}=\text{u}+\text{rand}\left(\text{l}-\text{u}\right)$$

In Equation 2 above, l and u represent the lower and upper bounds, respectively, and r and represents the random number in [0,1] . The fitness solution $\vec{X^{l}}$ is chosen to create the matrix known as Elite, and its mathematical formula is given in Equation 3:


(3) $$\begin{array}{c} \text{Elite}=[{\text{X}}_{1,1}^{\text{l}}\;{\text{X}}_{1,2}^{\text{l}}\;\ldots \;{\text{X}}_{1,\text{d}}^{\text{l}}\;{\text{X}}_{2,1}^{\text{l}}\;{\text{X}}_{2,2}^{\text{l}}\;\ldots \;{\text{X}}_{2,\text{d}}^{\text{l}}\;\vdots \;\vdots \;\\ {\text{X}}_{\text{n},1}^{\text{l}}\;\vdots \;\vdots \;{\text{X}}_{\text{n},2}^{\text{l}}\;\vdots \;\vdots \;\cdots \;\vdots \;\vdots \;{\text{X}}_{\text{n},\text{d}}^{\text{l}}\;]\text{n}\times \text{d}\; \end{array}$$

In Equation 3 above, the elite matrix includes the dimension [n,d] , d represents the search agents, and *n* represents the count of problem dimensions. Another matrix is known as prey is developed with the correct dimensions of Elite, and its mathematical formula is given in Equation 4.


(4) $$\begin{array}{c} \text{Prey}=[{\text{X}}_{1,1}\;{\text{X}}_{1,2}\;\cdots \;{\text{X}}_{1,\text{d}}\;{\text{X}}_{2,1}\;{\text{X}}_{2,2}\;\cdots \;{\text{X}}_{2,\text{d}}\;\vdots \;\vdots \\ \;{\text{X}}_{\text{n},1}\;\vdots \;\vdots \;{\text{X}}_{\text{n},2}\;\vdots \;\vdots \;\cdots \;\vdots \;\vdots \;{\text{X}}_{\text{n},\text{d}}\;]\text{n}\times \text{d} \end{array}$$

The process of MPA is separated to three phases which depended on the variance in velocity ratio among the prey and predator.

#### 3.3.2 Exploration phase—predator moving faster than the prey

While a prey is quicker than a predator, a predator’s optimal strategy remains unchanged. The exploration is much more significant in the first third of iterations. The prey location is updated through step size, and its mathematical formula is given in Equation 5,


(5) $$\vec{\text{stepsiz}{\text{e}}_{\text{i}}}\;=\;{\vec{\text{R}}}_{\text{B}}\;⊗\;\left({\vec{\text{Elite}}}_{\text{i}}-{\vec{\text{R}}}_{\text{B}}\;⊗\;{\vec{\text{Prey}}}_{\text{i}}\right),\;\text{i}=1,\;\ldots ,\;\text{n}$$

In Equation 5 above, $R_{B}$ represents the random number vector. The mathematical formula for the new position update is given in Equation 6:


(6) $${\vec{\text{Prey}}}_{\text{i}}\;=\;{\vec{\text{Prey}}}_{\text{i}}+0.5\cdot \vec{\text{N}}⊗{\vec{\text{stepsize}}}_{\text{i}}$$

In above Equation 6 above, $\vec{N}$ represents the random vector in the range of [0,1].

#### 3.3.3 Exploitation vs. exploration phase—the predator and prey moving at the same rate

While the prey and the predator move at similar speeds, they both prowl for prey. This phase occurs in an intermediate phase of the optimization process, where exploration attempts are temporarily shifted to exploitation. It is essential to balance both exploitation and exploration. As a result, half of a population is utilized for exploration and the next half is utilized for exploitation. In this phase, the predator is in exploration when a prey is in exploitation. The new location for the initial half of a population is updated, and its mathematical formula is given in Equations 7, 8.


(7) $$\vec{\text{stepsiz}{\text{e}}_{\text{i}}}\;=\;{\vec{\text{R}}}_{\text{L}}\;⊗\;\left({\vec{\text{Elite}}}_{\text{i}}-{\vec{\text{R}}}_{\text{L}}\;⊗\;{\vec{\text{Prey}}}_{\text{i}}\right),\;\text{i}=1,\;\ldots ,\frac{\text{n}}{2}$$

(8) $${\vec{\text{Prey}}}_{\text{i}}\;=\;{\vec{\text{Prey}}}_{\text{i}}+0.5\cdot \vec{\text{N}}\;⊗\;{\vec{\text{stepsize}}}_{\text{i}}$$

In Equation 7 above, ${\vec{R}}_{L}$ represents the random number vector depending on the Levy distribution. The new location for the next half of a population is updated, and its mathematical formula is given as Equations 9 and 10:


(9) $$\vec{\text{stepsiz}{\text{e}}_{\text{i}}}\;=\;{\vec{\text{R}}}_{\text{B}}\;⊗\;\left({\vec{\text{R}}}_{\text{B}}\;⊗\;{\vec{\text{Elite}}}_{\text{i}}-{\vec{\text{Prey}}}_{\text{i}}\right),\;\text{i}=\frac{\text{n}}{2},\;\ldots ,\;\text{n}$$

(10) $${\vec{\text{Prey}}}_{\text{i}}\;=\;{\vec{\text{Elite}}}_{\text{i}}+0.5\cdot \text{A}\;⊗\;{\vec{\text{stepsize}}}_{\text{i}}$$

In Equation 9 above, A represents the control parameter, and its mathematical formula is given as Equation 11:


(11) $$\text{A}={\left(1-\frac{\text{Iter}}{\text{MaxIter}}\right)}^{\left(2\frac{\text{Iter}}{\text{Maxlter}}\right)}\;$$

Exploitation phase—prey moving faster than the predator.

#### 3.3.4 Prey moving faster than predator (exploitation)

This phase occurred in a final phase of the optimization process and is integrated with high capability for the exploitation phase. The prey position is updated, and its mathematical formula is given in Equations 12, 13:


(12) $$\vec{\text{stepsiz}{\text{e}}_{\text{i}}}\;=\;{\vec{\text{R}}}_{\text{L}}⊗\left({\vec{\text{R}}}_{\text{L}}\;⊗\;{\vec{\text{Elite}}}_{\text{i}}-{\vec{\text{Prey}}}_{\text{i}}\right),\;\text{i}=1,\;\ldots ,\;\text{n}$$

(13) $${\vec{\text{Prey}}}_{\text{i}}\;=\;{\vec{\text{Elite}}}_{\text{i}}+\;0.5\cdot \text{A}\;⊗\;{\vec{\text{stepsize}}}_{\text{i}}$$

In the predation process, fish aggregation devices (FADs) fall into local optima, so the longer jumps are utilized for avoiding local optimal stagnation. The mathematical formula for jumping mode is given in Equation 14:


(14) $${\vec{\text{Prey}}}_{\text{i}}\;=\;\{\begin{array}{c} {\vec{\text{Prey}}}_{\text{i}}+\text{C}F\left[{\vec{\text{X}}}_{\text{m}i\text{n}}+\vec{\text{R}}⊗\left({\vec{\text{X}}}_{\text{m}a\text{x}}-{\vec{\text{X}}}_{\text{m}i\text{n}}\right)\right]⊗\vec{\text{U}}\;\;\text{i}f\;\text{r}\leq \text{F}A\text{D}s\;\\ {\vec{\text{P}re\text{y}}}_{\text{i}}+\left[\text{F}A\text{D}S\left(1-\text{r}\right)+\text{r}\right]\left({\vec{\text{P}r\text{e}y}}_{\text{r}1}-{\vec{\text{Prey}}}_{\text{r}2}\right)\;\;\text{i}f\;\text{r}>\text{FADs} \end{array}$$

In Equation 14 above, the value of FADs is 0.2, r represents the random number in the range of [0,1], ${\vec{X}}_{max}$ and the ${\vec{X}}_{min}$ represents the upper and lower limits of prey location, respectively, $\vec{U}$ represents the binary vector that has zeros and ones, and ${\vec{Prey}}_{r1}$ and ${\vec{Prey}}_{r2}$ represent the random prey locations.

#### 3.3.5 Random spiral flight strategy

By enhancing the traditional spiral search, the searchability of MPA is improved by changing the spiral search factor randomly. In the update stage, particularly in high-speed ratio scenarios, the RSFS strategy is implemented to enhance the position update flexibility of the chase search agent, balancing the global and local search space of MPA. The mathematical formula for the random spiral position update strategy is given in Equation 15:


(15) $${\vec{|\text{Prey}}}_{i}|={\vec{\text{Prey}}}_{\text{i}}+{\text{e}}^{\left(z\times \text{L}\right)}\times \text{c}o\text{s}\left(2\times \text{π}\times \text{L}\right)\times \left|{\vec{\text{E}l\text{i}t\text{e}}}_{\text{i}}-{\vec{\text{Prey}}}_{\text{i}}\right|\;$$

In Equation 15, z is a random spiral exploration factor, and its mathematical formula is given in Equation 16.


(16) $$\text{z}={\text{e}}^{\left(\text{k}\times \text{cos}\left(\text{π}\times \left(1-\frac{\text{Iter}}{\text{M}a{\text{x}}_{\text{Iter}}}\right)\right)\right)}\;$$

In Equation 16 above, k represents random spiral step length. **Table 1** presents the selected features from the PIMA and LMCH datasets.

**Table 1:** Selected features from PIMA and LMCH datasets.

Datasets	Selected features
PIMA	Glucose, BMI, age, diabetes, pedigree function, and insulin
LMCH	HBA1C, blood sugar level, BMI, age and LDL/HDL ratio

The RSFS-MPA efficiently selects features with strong clinical and statistical relevance such as BMI, age, LDL, blood sugar, and HBA1C. The features that are eliminated include gender, skin thickness, triglycerides, total cholesterol, and creatinine ratio, which show a weak correlation with diabetes labels.

### 3.4 Classification using Skip-GRU with GC approach

**Figure 4:**
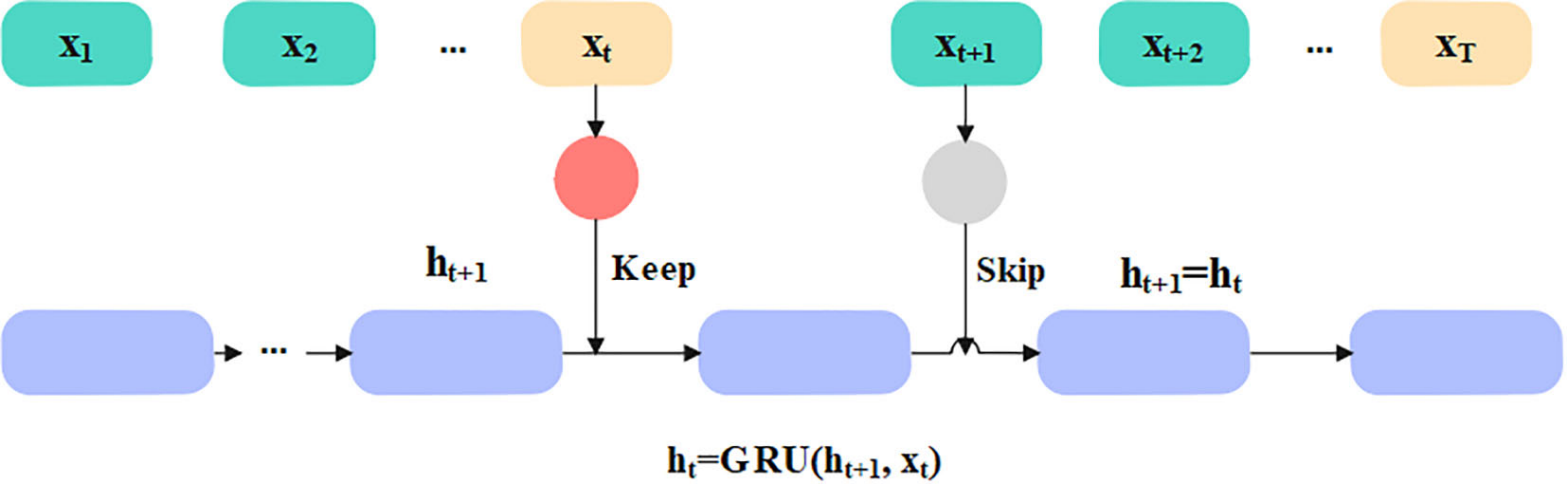
Architecture of Skip-GRU network.

The selected appropriate features are given as input to the classification phase to predict diabetes effectively. In this article, the Skip-GRU with GC is used to predict diabetes effectively. The Skip-GRU method eliminates inappropriate data, resulting in a much-streamlined process which leads to many correct predictions (33). The GC approach is used during the training process of Skip-GRU which helps to mitigate the exploding gradients issue. The parameters of Skip-GRU approach are 128 hidden size, 0.3 dropout rate, 0.1 weight decay, 0.0001 learning rate, and Adam optimizer. The Skip-GRU with GC approach has three phases like Skip network, GRU network, and GC method. The architecture of the Skip-GRU network is presented in **Figure 4**.

#### 3.4.1 Skip network

The Skip network measures the jump probability before the features are fed into the GRU network, which determines the data to be skipped and prevents much significance data in GRU in accordance with measured jump probability. This network is dependent on standard GRU. The input sequence is represented as $x_{1},\;x_{2},\;\ldots ,\;x_{T}$ . Before inputting a selected feature into the GRU network, it needs to be inputted into two layers such as multi-layer perceptron (MLP), and the jump probability distribution is measured through perceptron. The mathematical formula for jump probability calculation is given in Equations 17 and 18.


(17) $${\text{S}}_{\text{t}}=\text{RELU}\left({\text{W}}_{1}\text{S}+{\text{b}}_{1}\right)$$

(18) $${\text{π}}_{\text{t}}=\text{softmax}\left({\text{W}}_{2}{\text{S}}_{\text{t}}+{\text{b}}_{2}\right)$$

In Equations 17 and 18, $W_{1},\;W_{2},\;b_{1}$ , and $b_{2}$ represent the weights and biases, $S_{t}$ represents state of hidden state, and $\pi_{t}$ is the probability. In Skip-GRU architecture, every input feature is integrated with jump gate that calculated probability, representing whether a feature should be processed or skipped. In training, features with high jump probabilities are retained for GRU processing, representing its relevance in prediction. Features with less probabilities are ignored, which minimized noise and enhanced the model’s interpretability.

#### 3.4.2 GRU network

The GRU is the variant of recurrent neural network (RNN) which is a majorly utilized gated recurrent network. The GRU captured the long-term dependencies in diabetes, unlike the long short-term memory (LSTM) and RNN which have memory issues and are unable to capture the long-term dependencies completely. The GRU network has two gates: an update and a reset gate. The reset gate controls how input information is associated with prior memory, and the update gate determines how much prior memory is stored to the present time step. The standard GRU learns every feature and utilizes the update function to refresh the hidden state. The mathematical formula for gates in the GRU network is given in Equations 19-22:


(19) $${\text{r}}_{\text{t}}=\text{σ}\left({\text{W}}_{\text{r}}\cdot \left[{\text{h}}_{\text{t}-1},\;{\text{x}}_{\text{t}}\right]\right)$$

(20) $${\text{z}}_{\text{t}}=\text{σ}\left({\text{W}}_{\text{z}}\cdot \left[{\text{h}}_{\text{t}-1},\;{\text{x}}_{\text{t}}\right]\right)$$

(21) $${\tilde{\text{h}}}_{\text{t}}=\text{tanhtanh}\;\left(\text{W}\cdot \left[{\text{r}}_{\text{t}}\times {\text{h}}_{\text{t}-1},\;{\text{x}}_{\text{t}}\right]\right)$$

(22) $${\text{h}}_{\text{t}}=\left(1-{\text{z}}_{\text{t}}\right)\times {\text{h}}_{\text{t}-1}+{\text{z}}_{\text{t}}\times {\tilde{\text{h}}}_{\text{t}}\;$$

In Equations 18-22 above, $W_{r},\;W_{z}$ , and W represent various weight matrices, the $x_{t}$ represents the input data of the present moment, $h_{t-1}$ represents the hidden state of the prior moment, $r_{t}$ represents reset gate, $z_{t}$ represents update gate, and $h_{t}$ represents the output of cell. The value of skip probability $\pi_{t}$ determines the feature fed to GRU network, and its threshold value is chosen as 0.5. While $\pi_{t}<0.5$ , the feature is skipped, and the hidden layer is not updated. The mathematical formula for the hidden state is given in Equation 23:


(23) $${\text{h}}_{\text{t}}={\text{h}}_{\text{t}-1}$$

$\pi_{t}>0.5$ means that the feature is significant for classification results and fed into the GRU network. Currently, GRU’s hidden state is updated by using Equations 19-22.

#### 3.4.3 Gradient clipping

In this article, the GC method is used to clip the excessive gradient value to the threshold value for mitigating the gradient explosion during training. The threshold of gradient clipping is set to 1. Consider the function f(X;θ) computed on data X and parameterized by θ and λ that represent the learning rate; the gradient descent updates at t iteration of the present parameters $\theta_{t-1}$ to $\theta_{t}$ , and the mathematical formula is given as Equation 24:


(24) $${\text{θ}}_{\text{t}}={\text{θ}}_{\text{t}-1}-\text{λ}\nabla_{\text{θ}}\text{f}\left(\text{X};{\text{θ}}_{\text{t}-1}\right)$$

The GC enforces the upper bound on θ updation through positioning the maximum on the gradient norm. The mathematical formula is given in Equations 25 and 26:


(25) $${\text{θ}}_{\text{t}}={\text{θ}}_{\text{t}-1}-\text{λ}{\text{h}}_{\text{c}}\nabla_{\text{θ}}\text{f}\left({\text{X}}_{\text{t}};{\text{θ}}_{\text{t}-1}\right)$$

(26) $${\text{h}}_{\text{c}}=\left\{\frac{{\text{η}}_{\text{c}}}{\left\|\nabla_{\text{θ}}\text{f}\left({\text{X}}_{\text{t}};{\text{θ}}_{\text{t}-1}\right)\right\|},\;1\right\}$$

In the equations above, $\eta_{c}$ represents the clipping values. This clipping process is also known as clip by norm, not clip by value, where separate gradient vector values are clipped if it is extended to the threshold value. Here the whole gradients are scaled whether the norm of gradient extends the threshold. In GC, $\eta_{c}$ selection is significant. Whether it is too large, then the gradient norm is smaller and clipping is not employed. If it is too low, then the step size considered through the network is too little. The classification using Skip-GRU network is presented in **Algorithm 1**.

Algorithm 1 **Process of Skip-GRU.**

**Input**: Selected features
**Output**: predicted result
**for** feature 
S
 in 
T
 **do**
  
$S_{t}=RELU\left(W_{1}S+b_{1}\right)$

  
$\pi_{t}=softmax\left(W_{2}S_{t}+b_{2}\right)$

  **if** 
$\pi_{t}<0.5$
 then
   skip this feature
  **else**
   fed 
x
 to GRU network
  **end if**
**end for**

## 4 Experimental analysis

The proposed Skip-GRU with GC approach is simulated with a Python 3.8 environment, and the required system configurations are 8 GB RAM, Windows 10 (64 bit), and i5 processor. The metrics taken to evaluate the performance of Skip-GRU with the GC approach are accuracy, sensitivity, precision, F1-score, and specificity on PIMA and LMCH datasets. The mathematical formula for metrics is given in Equations 27-31.

Accuracy—This is the ratio of the whole number of accurate predictions to the whole number of predictions, and its mathematical formula is given in Equation 21.


(27) $$\text{Accuracy}=\;\frac{\text{TP}+\text{TN}}{\text{TP}+\text{TN}+\text{FP}+\text{FN}}\times 100$$

Sensitivity—This is the ratio of patients with diabetes (positive instances) who are accurately identified as diabetic, and it is calculated as a proportion of true positives (TP) to the sum of TP and false negatives (FN). The mathematical formula for sensitivity is given as Equation 28:


(28) $$\text{Sensitivity}=\frac{\text{TP}}{\text{TP}+\text{FN}}\times 100$$

Specificity—This is the ratio of patients without diabetes (negative instances) who are accurately identified as non-diabetic, and it is calculated as the proportion of true negatives (TN) to the sum of TN and false positives (FP). The mathematical formula for specificity is given as Equation 29:


(29) $$\text{Specificity}=\frac{\text{TN}}{\text{TN}+\text{FP}}\times 100$$

Precision—This is the ratio of patients with diabetes, positive instances, who are accurately identified as diabetic from whole diabetic patients, and it is executed as a proportion of TP to the sum of TP and FP. The mathematical formula for precision is given as Equation 30:


(30) $$\text{Precision}=\frac{\text{TP}}{\text{TP}+\text{FP}}\times 100$$

F1-score—This is the average of precision and sensitivity, and it considers the value of both FP and FN. It is given as Equation 31:


(31) $$\text{F}1-\text{score}=\frac{2\text{TP}}{2\text{TP}+\text{FP}+\text{FN}}\times 100\;$$

**Table 2** presents the explored ranges and final selected values for every hyperparameter. These values are chosen based on empirical testing using the PIMA and LMCH datasets. A smaller learning rate of 0.001 stabilized the training and improved the convergence. It ensures smooth and well-controlled weight updates when larger learning rates resulted in oscillations and suboptimal convergence. A hidden unit size of 128 provides better trade-off and offers effective learning ability and improved generalization ability. Fewer hidden units lead to underfitting, while a larger number of hidden units causes overfitting. A dropout rate of 0.3 prevents overfitting by randomly deactivating neurons in training, thereby enhancing generalization. The higher dropout rates introduced excessive regularization, which degrades performance through limiting the model’s ability to learn complex feature interactions. A weight decay value of 0.1 provides strong regularization that penalized large weight magnitudes and effectively constrains model complexity, thereby reducing overfitting. Lower weight decay values provide ineffective regularization, while larger values cause underfitting through the excessive restriction of the model’s learning ability. A batch size of 32 offers a better trade-off between stable gradient estimates and memory efficacy. Smaller batch sizes introduce a high variance in gradient updates, causing noisy and unstable training, while larger batches reduce gradient noise, but slows down convergence and increases the generalization error because of less weight updates. A gradient clipping threshold of 1.0 effectively mitigates the exploding gradient problem in the training process of the Skip-GRU model. It constrains the norm of gradients in a controlled range and provides stable and consistent parameter updates. Lower threshold values reduce the gradient flow, leading to slow learning and poor convergence, while higher values fail to prevent large updates, resulting in training instability.

**Table 2:** Explored ranges and final selected values for every hyperparameter.

Hyperparameter	Explored values	Final selected values
Learning rate	[0.0001, 0.001, 0.005, 0.001]	0.0001
Hidden units	[64, 96, 128, 160, 192]	128
Dropout rate	[0.1, 0.2, 0.3, 0.4]	0.3
Weight decay	[0.0, 0.01, 0.05, 0.1]	0.1
Batch size	[16, 32, 64]	32
Gradient clipping Threshold	[0.5, 1.0, 1.5, 2.0]	1.0

In **Table 3**, the performance of the feature selection algorithm is evaluated on the PIMA and LMCH datasets with different metrics. The different feature selection algorithms considered for evaluating the developed RSFS-MPA are the reptile search algorithm (RSA), crow search algorithm (CSO), crayfish optimization algorithm (COA), and traditional MPA. The RSFS is incorporated with MPA for the feature selection process, which improves the search ability and effectively balances the local and global processes of traditional MPA. By using this strategy, the MPA effectively searches for the best feature subset and chooses the best features for classification. The developed RSFS-MPA achieved 97.84% sensitivity, 97.35% precision, 97.59% F1-score, 98.23% accuracy, and 97.61% specificity on the PIMA dataset and 97.21% sensitivity, 97.46% precision, 97.33% F1-score, 97.65% accuracy, and 97.53% specificity on the LMCH dataset.

**Table 3:** Performance of the feature selection process.

Algorithms	Accuracy (%)	Sensitivity (%)	Precision (%)	F1-score (%)	Specificity (%)
PIMA dataset					
RSA	96.53	96.38	96.05	96.21	96.27
CSA	96.92	96.62	96.31	96.46	96.47
COA	97.32	97.07	96.77	96.91	96.91
MPA	97.87	97.52	97.06	97.28	97.37
RSFS-MPA	98.23	97.84	97.35	97.59	97.61
LMCH dataset					
RSA	96.09	95.87	95.42	95.64	95.73
CSA	96.37	96.04	95.79	95.91	96.18
COA	96.78	96.37	96.06	96.21	96.64
MPA	97.24	96.85	96.57	96.70	97.17
RSFS-MPA	97.65	97.21	97.46	97.33	97.53

In **Table 4**, the performance of the classifier is evaluated on the PIMA and LMCH datasets with actual features based on different metrics. The different classifiers considered for evaluating the Skip-GRU with GC approach are recurrent neural network (RNN), long short-term memory (LSTM), GRU, and Skip-GRU. The proposed Skip-GRU with GC achieved 96.25% sensitivity, 95.87% precision, 96.05% F1-score, 96.71% accuracy, and 95.46% specificity on the PIMA dataset and 95.19% sensitivity, 95.32% precision, 95.25% F1-score, 95.25% accuracy, and 95.43% specificity on the LMCH dataset.

**Table 4:** Performance of Skip-GRU with GC approach with actual features.

Classifiers	Accuracy (%)	Sensitivity (%)	Precision (%)	F1-score (%)	Specificity (%)
PIMA dataset					
RNN	95.07	94.87	94.48	94.67	94.65
LSTM	95.44	95.14	94.77	94.95	94.93
GRU	95.88	95.52	95.29	95.40	95.45
Skip-GRU	96.32	96.06	95.54	95.79	95.77
Skip-GRU with GC	96.71	96.25	95.87	96.05	95.46
LMCH dataset					
RNN	93.78	93.52	93.16	93.33	93.38
LSTM	94.27	93.93	93.64	93.78	93.81
GRU	94.85	94.52	94.12	94.31	94.38
Skip-GRU	95.18	94.88	94.35	94.61	94.86
Skip-GRU with GC	95.76	95.19	95.32	95.25	95.43

In **Table 5**, the performance of the classifier is evaluated on the PIMA and LMCH datasets after feature selection based on different metrics. The different classifiers considered for evaluating the Skip-GRU with GC approach are RNN, LSTM, GRU, and Skip-GRU. Here the relevant features from the whole feature subset are selected by using the RSFS-MPA feature selection technique. By eliminating the irrelevant features, only the relevant features are fed into the classification process and help to enhance the classification performance. By using the Skip-GRU approach, unwanted features were skipped and only the significant features for classification were fed. Then, the GC technique is used during the training process of Skip-GRU, which helps to mitigate the issue of exploding gradients and enhances the process of Skip-GRU for diabetes prediction. The RFFS-MPA effectively filters out inappropriate and redundant features by minimizing noise. This selected feature refinement resulted in a much focused training process and minimized the overfitting issue, which is especially significant in medical datasets that contain correlated and low-variance features. The Skip-GRU model enhances the performance through dynamically ignoring irrelevant inputs by the jump probability mechanism. This enables a model to adaptively control data flow and allows essential temporal inputs to influence the hidden states. From the results, Skip-GRU consistently outperformed existing models like RNN, LSTM, and GRU across the entire evaluation metrics. The incorporation of GC to Skip-GRU model provides stability in the training process through gradient magnitudes, which efficiently addresses the exploding gradient issue. This is particularly essential while dealing with high-dimensional data, as the uncontrolled gradients cause unstable updates and degrading performance. The proposed Skip-GRU with GC approach achieved 97.84% sensitivity, 97.35% precision, 97.59% F1-score, 98.23% accuracy, and 97.61% specificity on the PIMA dataset and 97.21% sensitivity, 97.46% precision, 97.33% F1-score, 97.65% accuracy, and 97.53% specificity on the LMCH dataset.

**Table 5:** Performance of Skip-GRU with GC approach after feature selection.

Classifiers	Accuracy (%)	Sensitivity (%)	Precision (%)	F1-score (%)	Specificity (%)
PIMA dataset					
RNN	96.86	96.64	96.25	96.44	96.47
LSTM	97.09	96.88	96.47	96.67	96.62
GRU	97.54	97.23	96.87	97.04	96.78
Skip-GRU	97.83	97.49	97.15	97.31	97.32
Skip-GRU with GC	98.23	97.84	97.35	97.59	97.51
LMCH dataset					
RNN	96.08	95.89	95.32	95.60	95.32
LSTM	96.44	96.16	95.78	95.96	95.67
GRU	96.82	96.43	96.07	96.24	96.22
Skip-GRU	97.15	96.79	96.48	96.63	96.75
Skip-GRU with GC	97.65	97.21	97.46	97.33	97.17

In **Table 6**, the performance of Skip-GRU with the GC approach is evaluated based on k-fold validation. It splits the dataset into multiple subsets and uses every fold as a training and validation set in various iterations. Instead of training and testing on similar data, the k-fold cross-validation gives a much more reliable estimation of method performance on unseen data. The performance of Skip-GRU with GC approach is evaluated for k-values of 2, 3, 4, 5, and 6. In that, K = 5 has achieved the highest values when compared to other k-fold values and provides better balance among training and validation coverage. This analysis is essential to validate the model’s robustness, stability, and generalization ability over various training–testing splits. In K-fold cross-validation, the dataset is divided into K folds and the model is trained K times, every time using K-1 folds for training and remaining fold for testing. This ensures that each instance in the dataset is utilized for training and validation and outcomes are averaged to overcome variance because of random data splits. The results show that Skip-GRU with GC model provides a consistent performance over different K-fold configurations. The less fluctuation across folds determines that the model is not sensitive to data partitioning.

**Table 6:** K-fold validation for Skip-GRU with GC approach.

K-fold values	Accuracy (%)	Sensitivity (%)	Precision (%)	F1-score (%)	Specificity (%)
PIMA dataset					
K=2	96.86	96.39	96.17	96.27	95.60
K=3	97.15	96.75	96.43	96.58	96.34
K=4	97.58	97.28	96.87	97.07	96.73
**K=5**	**98.23**	**97.84**	**97.35**	**97.59**	**97.51**
K=6	97.94	97.53	97.06	97.29	97.22
LMCH dataset					
K=2	96.18	95.89	95.57	95.72	95.84
K=3	96.57	96.25	96.03	96.13	96.02
K=4	97.07	96.77	97.01	96.88	96.45
**K=5**	**97.65**	**97.21**	**97.46**	**97.33**	**97.17**
K=6	97.34	97.09	97.22	97.15	96.85

The significance of K=5 as optimal fold in cross-validation due to its ability to balance model performance and evaluation stability, that consistently provides highest values across all metrics for PIMA and LMCH datasets. This shows that K=5 effectively captures data variability when reducing overfitting and underfitting, ensures generalization ability to unseen data.

### 4.1 Generalization analysis

To analyze the robustness and generalization ability of the model, we conducted external validation using the National Health and Nutrition Examination Survey (NHANES) (34) dataset. NHANES includes a large, demographic diverse population across different ethnicities, age groups, and socioeconomic backgrounds in the Unites States (US), making it an ideal benchmark for real-world performance assessment.

NHANES is the studies program developed to assess the nutritional and health status of children and adults in US. NHANES is the primary program of the National Center of Health Statistics (NCHS). NCHS is part of the Centers to Disease Control and Prevention (CDC) and has responsibility to produce primary and health statistics for the nation. The survey analyzes a nationally representative sample of 5,000 persons every year. These persons are positioned across countries, 15 of which are visited every year. The NHANES interview involves socioeconomic, dietary, demographic, and health-relevant questions. The examination includes dental, physiological measurements, medical, and laboratory tests through highly trained medical personnel.

The table presents a performance comparison of different neural architectures like RNN, LSTM, GRU, and Skip-GRU, with the proposed Skip-GRU with GC on the NHANES dataset. The Skip-GRU with GC offers better outcomes across all evaluation metrics. The incorporation of gradient clipping stabilizes the training process, minimizes gradient explosion, and enhances convergence. This results in significantly better sensitivity and specificity, which are essential to minimize false negatives and false positives in clinical diagnosis. The proposed model demonstrates a strong generalization ability on the NHANES dataset, validating its effectiveness across controlled datasets. **Table 7** presents a generalization analysis of Skip-GRU with GC model using the NHANES dataset.

**Table 7:** Generalization analysis of proposed Skip-GRU with GC model using NHANES dataset.

Classifiers	Accuracy (%)	Sensitivity (%)	Precision (%)	F1-score (%)	Specificity (%)
RNN	90.45	88.21	87.10	87.65	91.67
LSTM	92.18	90.34	89.50	89.92	93.25
GRU	93.12	91.76	90.62	91.18	94.20
Skip-GRU	94.88	93.31	92.40	92.85	95.78
Skip-GRU with GC	96.45	95.28	94.81	95.04	97.12

### 4.2 Interpretability analysis

Interpretability represents the ability to understand and explain the prediction of a model in a human-understandable manner. In clinical decision-making, interpretability is significant due to healthcare professional needs to trust, validate, and justify the results of the model. Without transparency, a highly accurate model will reject because of its black-box nature. To address this, in this manuscript, local interpretable model-agnostic explanations (LIME) for interpretability was used. The LIME process is through perturbing input data and learning the interpretable model locally around every prediction. The jump mechanism of Skip-GRU shows model interpretability through employing significance weights to input features. The features fed to GRU with those highlighted through LIME show its relevance. This provides global and local interpretability, which is crucial for clinical research. This process supports the things below:

- It identifies which features much influenced a particular prediction.
- It highlights the patient-specific risk factors which contribute to diabetes prediction.
- It provides clinicians with visual and intuitive explanations to support decision-making.

**Figure 5:**
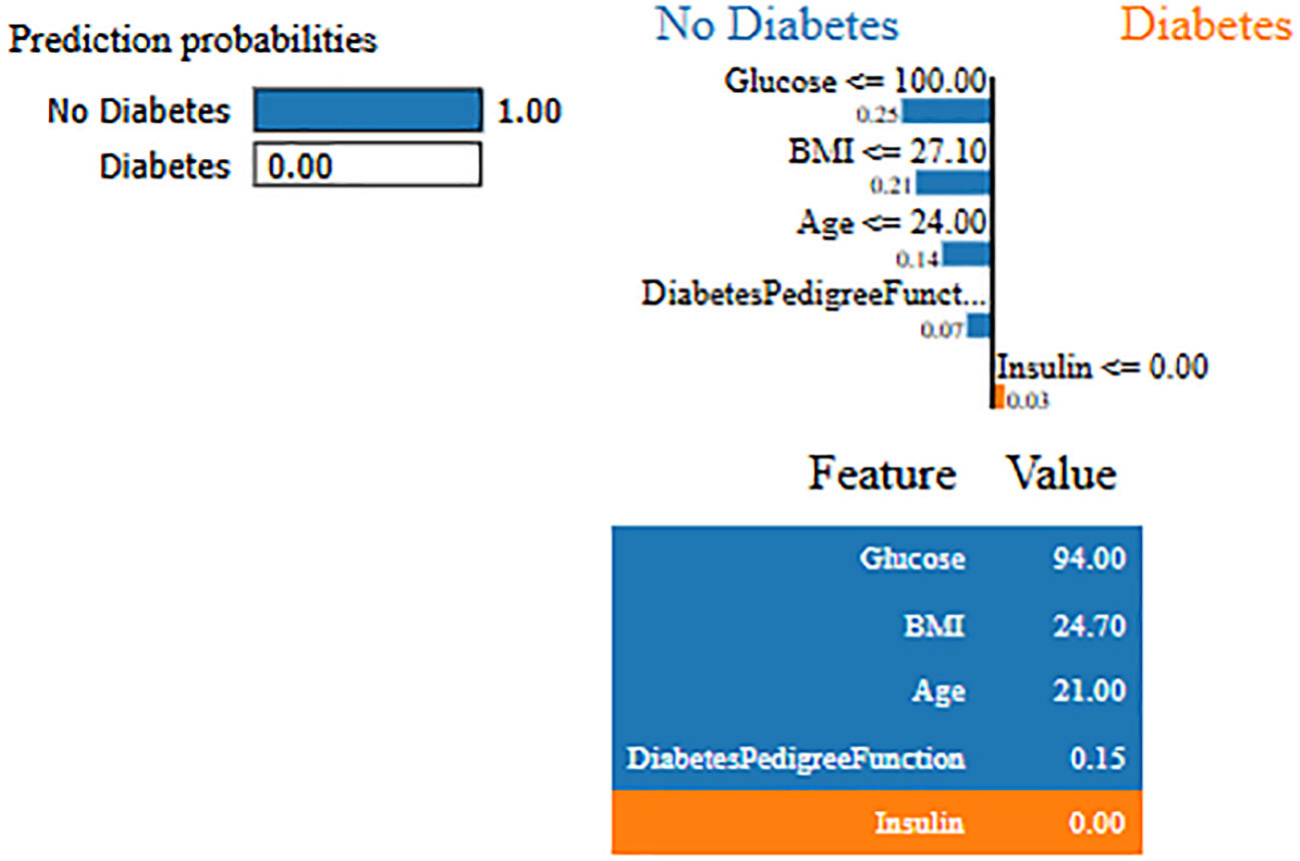
Interpretability of non-diabetes class for PIMA dataset.

**Figure 6:**
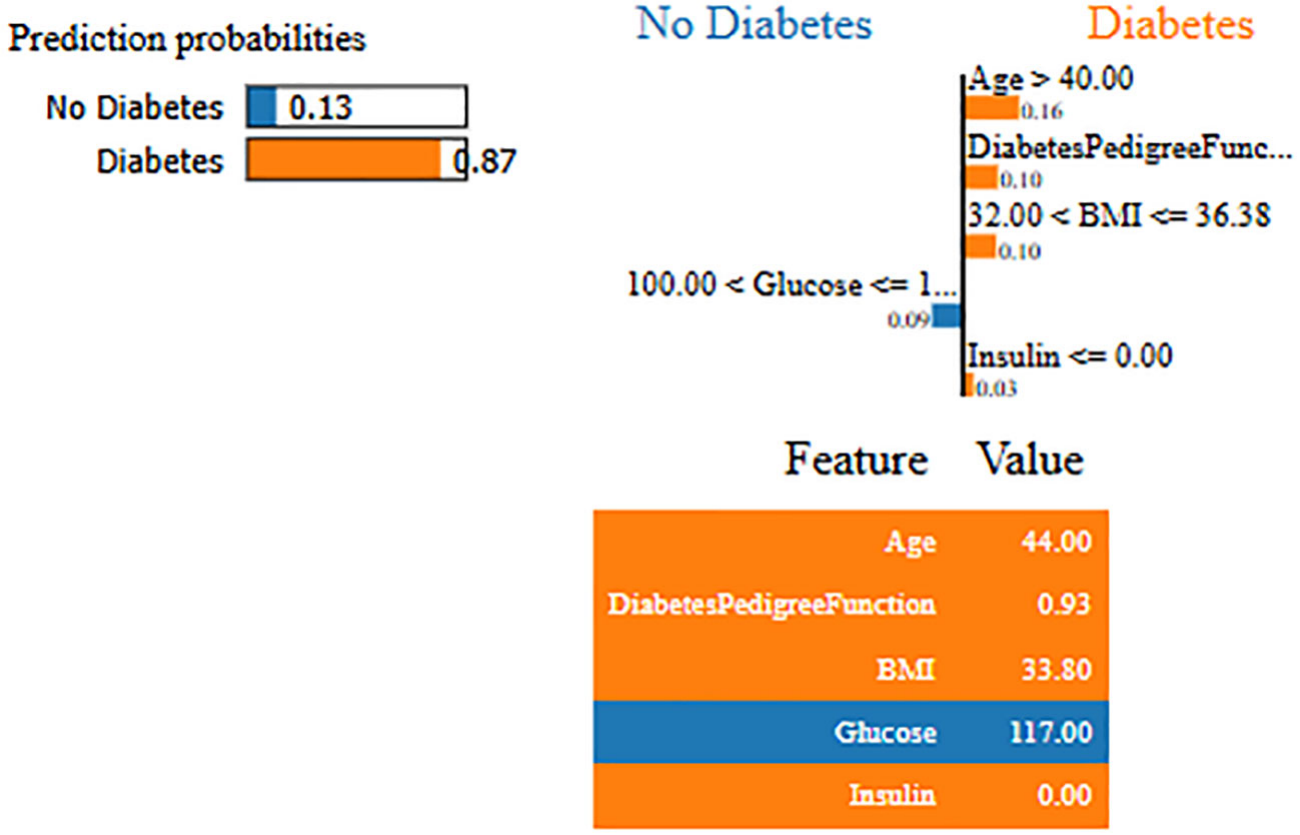
Interpretability of diabetes class for PIMA dataset.

**Figure 7:**
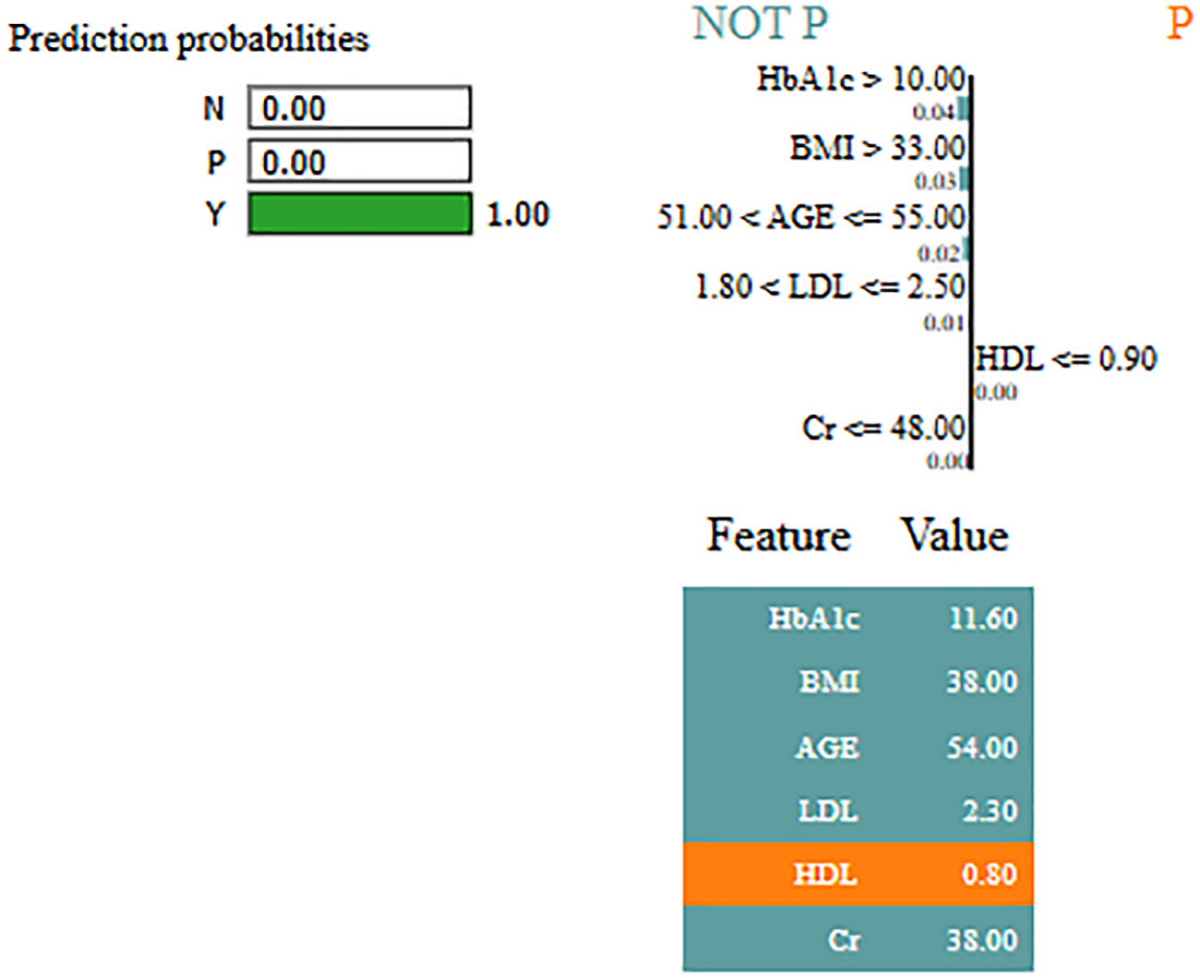
Interpretability of diabetes class for LIMCH dataset.

**Figure 8:**
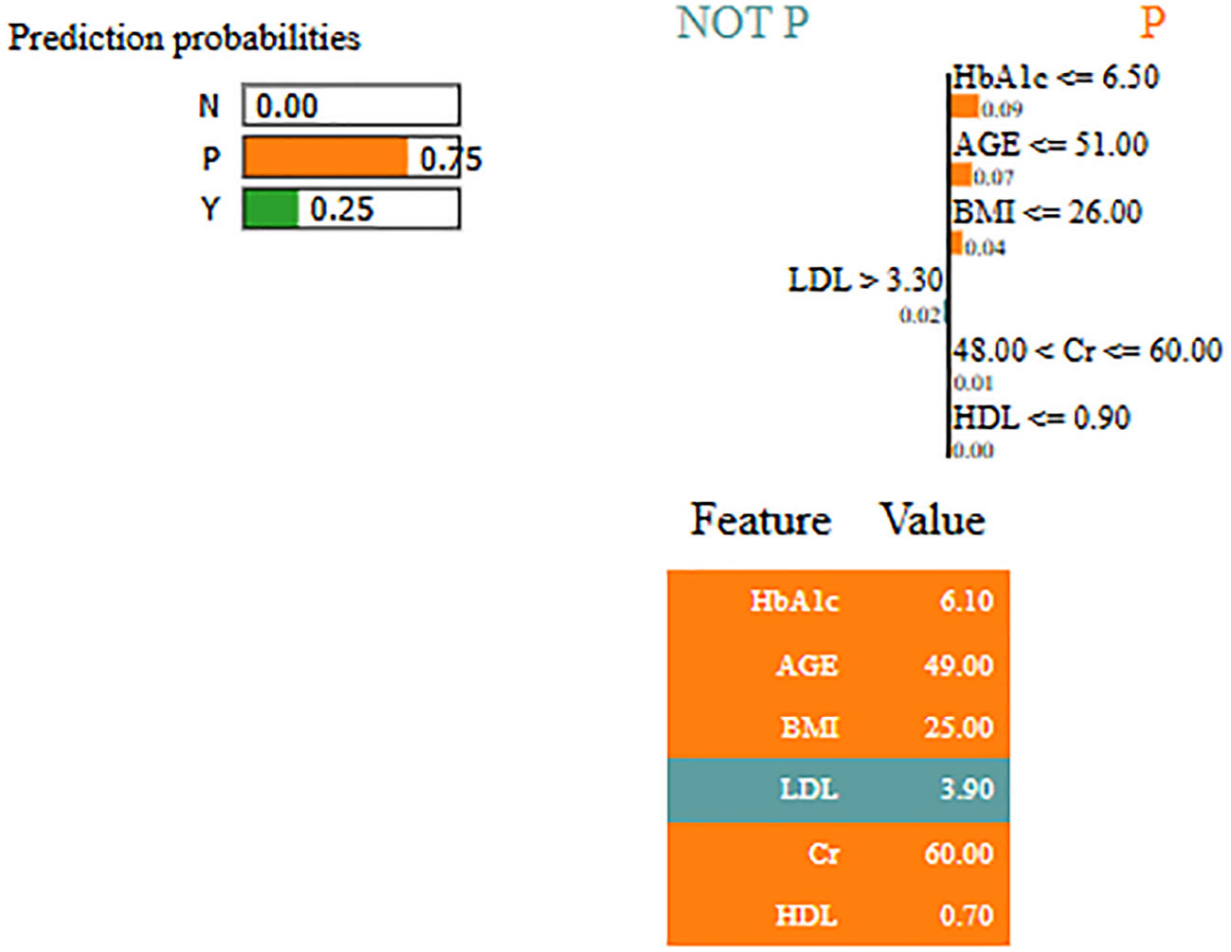
Interpretability of pre-diabetes class for LMCH dataset.

**Figure 9:**
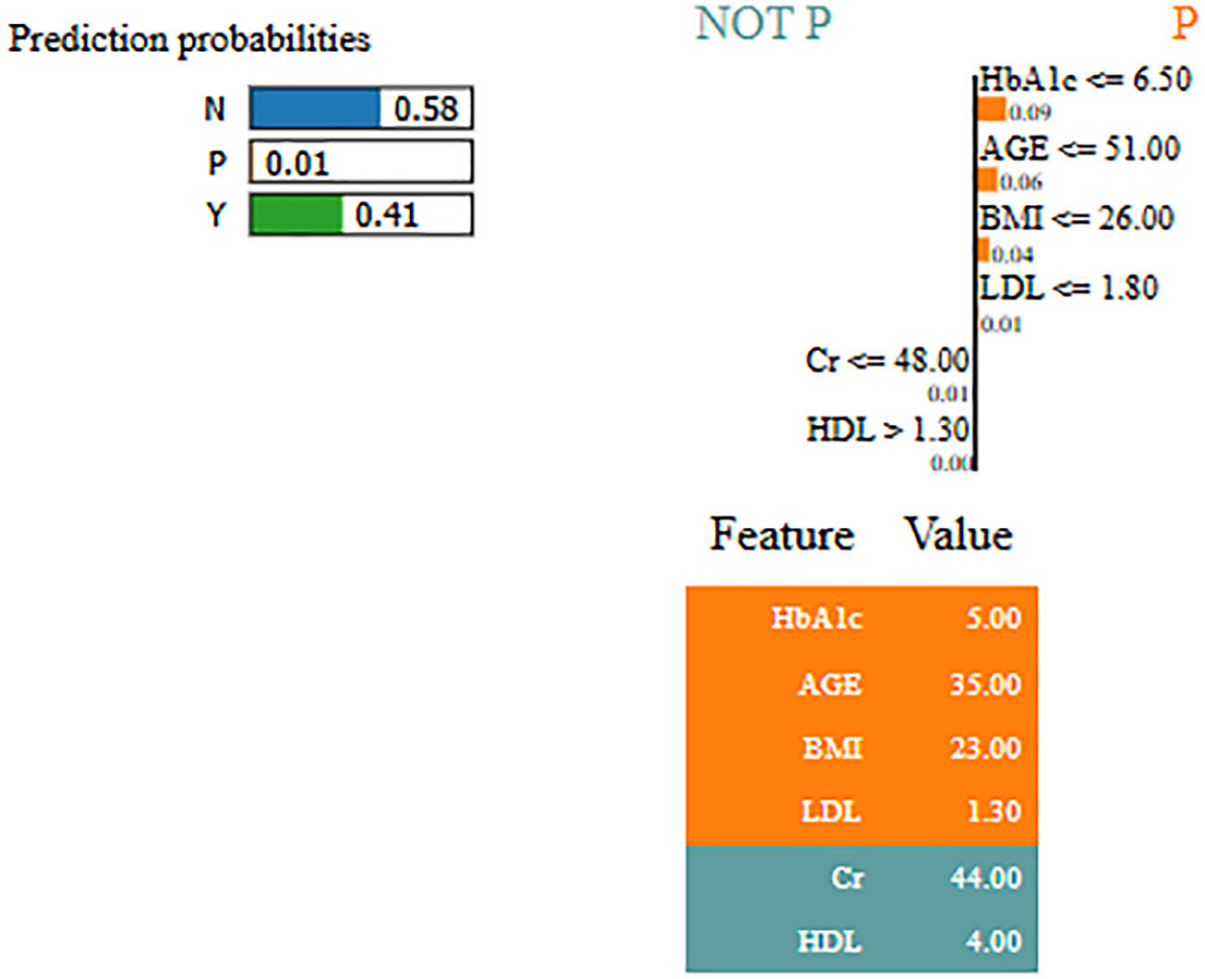
Interpretability of non-diabetes class for LMCH dataset.

By using LIME, it ensures that the model performs well statistically and provides meaningful insights as to why the prediction is made by maximizing their reliability. **Figures 5** and **6** present the interpretability of non-diabetes and diabetes classes using the LIME model for the PIMA dataset, respectively. **Figures 7**–**9** present the diabetes, pre-diabetes, and non-diabetes classes interpretability for the LMCH dataset, respectively.

### 4.3 Comparative analysis

In **Table 8**, the performance of Skip-GRU with GC approach is compared with existing techniques like Extra Tree classifier (20), 2GDNN (21), SVM with integrated kernel (22), and seven-layered deep CNN (23) on the PIMA and LMCH datasets. In this article, the RSFS–MPA is used for the feature selection phase which selects the appropriate features by eliminating the irrelevant features and helps to enhance the classification performance. The RSFS is incorporated into traditional MPA, which improves the search ability of MPA for the feature selection process. Then, these selected relevant features are inputted to Skip-GRU with the GC method. Here the significant features are allowed, and the unnecessary features are skipped. The GC technique is used during the Skip-GRU training process, which helps to mitigate the exploding gradients issue in the training process. These processes enhance the classification performance of Skip-GRU with the GC approach and effectively predict diabetes. The proposed Skip-GRU with GC approach achieved 98.23% accuracy on the PIMA dataset and 97.65% accuracy on the LMCH dataset.

**Table 8:** Comparative analysis of Skip-GRU with GC approach.

Datasets	Methods	Accuracy (%)	Precision (%)	Recall (%)	F1-score (%)
PIMA	Extra Tree classifier (20)	89	NA	NA	NA
	2GDNN (21)	97.25	97.34	97.24	97.26
	SVM with integrated kernel (22)	85.5	NA	NA	85.2
	7-layer-deep CNN (23)	88.38	83.33	NA	NA
	Proposed Skip-GRU with GC	98.23	97.84	97.35	97.59
LMCH	2GDNN (21)	97.33	97.28	97.33	97.27
	Proposed Skip-GRU with GC	97.65	97.21	97.46	97.33

NA, Not Available.

### 4.4 Discussion

This section analyzed the outcomes of the proposed Skip-GRU with GC method from the PIMA and LMCH datasets. The performance of Skip-GRU with GC method is evaluated using different optimization algorithms like RSA, CSO, COA, and traditional MPA. Additionally, it is evaluated with different classifiers like RNN, LSTM, GRU, and Skip-GRU. Moreover, the results are compared with existing techniques like Extra Tree classifier (20), 2GDNN (21), SVM with integrated kernel (22), and seven-layer-deep CNN (23) on the PIMA and LMCH datasets. In the result section, the performance of Skip-GRU with the GC method is evaluated using k-fold cross-validation on the PIMA and LMCH datasets. **Tables 1**–**3** present the accuracy, sensitivity, precision, specificity, and F1-score for RSA, CSO, COA, traditional MPA, RNN, LSTM, GRU, and Skip-GRU. By experimental outcomes from the PIMA and LMCH datasets, the developed Skip-GRU with GC approach effectively predicts diabetes with high accuracy. When compared with existing methods like Extra Tree classifier (20), 2GDNN (21), SVM with Integrated kernel (22) and 7-layered deep CNN (23), the developed Skip-GRU with GC approach performed well and obtained 98.23% accuracy on the PIMA dataset and 97.65% accuracy on the LMCH dataset. These existing approaches have limitations such as the values in the dataset not being scaled uniformly, not imputing the missing values, and then skipping the feature selection process. Certain ML approaches were unable to capture the long-term dependencies and did not address the issue of exploding gradients. To overcome these drawbacks from existing techniques, this article used the min–max normalization technique to scale the values uniformly, and PR technique is used to impute the missing values in the dataset. Then, the RSFS–MPA-based feature selection algorithm is developed to select the relevant features, and then for classification the Skip-GRU with GC approach was used. This approach captures the long-term dependencies and mitigates the issue of exploding gradients with the help of the GC method during the training process. These processes help the model to predict diabetes effectively with high performance and accuracy. The proposed Skip-GRU architecture filters out irrelevant inputs by jump probability evaluation, making it crucial to find which particular features are consistently chosen as relevant. The Skip network determines whether a feature should pass to GRU or be skipped depending on the calculated jump probability. Features with high jump probability are considered as relevant, while those with less values are skipped to minimize noise and model complexity. By incorporating jump probabilities in training and inference, the model tracks the relative importance of every feature over dataset and in individual predictions. The Skip-GRU with GC model obtained a higher performance on the PIMA dataset because of its relatively balanced class distribution and low-dimensional feature space, while the LMCH dataset presents higher challenges for model learning primarily because of class imbalance and increased variability in real-world clinical data, including missing values and complex inter-feature dependencies.

### 4.5 Limitations

Although the proposed Skip-GRU with GC model has been validated on three datasets such as PIMA, LMCH, and NHANES, potential population-specific bias still exists. While NHANES offers a broad and different representation of US individuals over various ethnic and age groups, it does not fully capture global variations in diabetes risk factors. Additionally, clinical, genetic, and lifestyle differences in populations from various regions (Africa, Asia, Latin America, and Southeast) will influence the model’s performance and generalization ability.

## 5 Conclusion

This research developed an effective DL-based approach for enhancing diabetes prediction performance using the PIMA and LMCH datasets. The processes involved in diabetes prediction are pre-processing the data using min–max normalization and PR and then the feature selection using the RSFS-MPA. Finally, the data is classified by using the Skip-GRU with GC approach. The RSFS used in traditional MPA enhanced the performance of traditional MPA for feature selection. This process eliminates the irrelevant features from the whole feature subset and selects only the relevant features and feeds them to the classification process. The Skip-GRU approach effectively classifies diabetes with high accuracy. The GC technique is introduced in the Skip-GRU approach during the training phase, which mitigates the exploding gradient issue and enhances the performance of diabetes prediction. The proposed Skip-GRU with GC approach achieved 98.23% accuracy on the IMA dataset and 97.65% accuracy on the LMCH dataset when compared to existing approaches like the Extra Tree classifier and seven-layer-deep CNN.

### 5.1 Future work

Future work will focus on multi-regional and real-world clinical datasets to improve the generalization ability across varied healthcare environments. This will address the potential distributional shifts in clinical data arising from regional, demographic, and systemic variations.

## Data Availability

Publicly available datasets were analyzed in this study. This data can be found here: https://www.kaggle.com/datasets/uciml/pima-indians-diabetes-databasehttps://pmc.ncbi.nlm.nih.gov/articles/PMC11098411/.
